# Shedding Light on the Effects of Moderate Acute Exercise on Working Memory Performance in Healthy Older Adults: An fNIRS Study

**DOI:** 10.3390/brainsci10110813

**Published:** 2020-11-03

**Authors:** Katharina Stute, Nicole Hudl, Robert Stojan, Claudia Voelcker-Rehage

**Affiliations:** 1 Institute of Human Movement Science and Health, Chemnitz University of Technology, 09126 Chemnitz, Germany; katharina.stute@hsw.tu-chemnitz.de (K.S.); nicole.hudl@hsw.tu-chemnitz.de (N.H.); robert.stojan@uni-muenster.de (R.S.); 2 Department of Neuromotor Behavior and Exercise, Institute of Sport and Exercise Sciences, University of Muenster, 48149 Muenster, Germany

**Keywords:** aging, cardiovascular exercise, cognition, executive functions, fronto-parietal network, neuroimaging

## Abstract

Numerous studies have reported the beneficial effects of acute exercise on executive functions. Less is known, however, about the effects of exercise on working memory as one subcomponent of executive functions and about its effects on older adults. We investigated the effects of acute moderate-intensity exercise on working memory performance, the respective cortical hemodynamic activation patterns, and the development and persistence of such effects in healthy older adults. Forty-four participants (*M*: 69.18 years ± 3.92; 21 females) performed a letter 2-back task before and at three time points after (post 15 min, post 30 min, and post 45 min) either listening to an audiobook or exercising (15 min; 50% VO_2_-peak). Functional near-infrared spectroscopy (fNIRS) was used to assess cortical hemodynamic activation and brain-behavior correlations in the fronto-parietal working memory network. Overall, we found no group differences for working memory performance. However, only within the experimental group, 2-back performance was enhanced 15 min and 45 min post-exercise. Furthermore, 15 min post-exercise frontal activation predicted working memory performance, regardless of group. In sum, our results indicate slight beneficial effects of acute moderate-intensity exercise on working memory performance in healthy older adults. Findings are discussed in light of the cognitive aging process and moderators affecting the exercise-cognition relationship.

## 1. Introduction

Numerous studies in the field of sport and exercise sciences have focused on the human aging process and examined the relationship between chronic exercise and cognition from a neuroscientific perspective [1,2]. More recently, researchers have become increasingly interested in how and to what extent a single bout of exercise (also referred to as acute exercise) affects cognitive performance in executive control tasks. Executive control is a concept that encompasses a number of processes, such as updating of information, shifting between tasks, and inhibiting responses and/or information [3,4]. The premise underlying acute exercise-executive control research is that physiological changes (e.g., increased cerebral blood flow) induced by acute exercise have the potential to improve executive control [5].

Meta-analytic evidence suggests that acute exercise might be particularly beneficial for older adults [6,7], probably due to compromised cognitive reserve and frontal lobe functioning and they thus have greater capacity for improvement in response to exercise compared to young adults [8]. Yet, age-related changes differ noticeably both inter- and intra-individually and across cognitive domains [9,10], and hence it is probably also the case with the effects of exercise. One executive function particularly sensitive to age-related decline is working memory [11,12,13]. Working memory is an important component underlying higher-level cognitive processes [9], such as language comprehension, learning, and reasoning, which requires simultaneous storage and processing of information [3]. Working memory performance decreases with age due to neural changes of the underlying brain networks [14,15]. Verbal working memory tasks (e.g., the letter n-back task) have been typically associated with a frontal left dominant neural activation pattern in young adults [16,17,18]. Yet, bilateral hemispheric recruitment of the prefrontal cortex (PFC) has been described to occur as an age-independent domain-general strategy to master increasing task demands [19]. Typically, older adults show a hemispheric asymmetry reduction of prefrontal activation, which is generally referred to as the hemispheric asymmetry reduction in older adults (HAROLD) model [20]. In addition to frontal brain regions, parietal brain regions are likewise involved in the human working memory network. Within the fronto-parietal working memory network [21], frontal regions are associated with the executive processing components [22]. These processing components have been reported to be more affected by the human aging process than the temporary storage components associated with parietal brain regions [18,23], where the integration of visuo-spatial and associative information takes place [24]. Similarly, evidence from neuroimaging studies supports the hypothesis that older adults show a relative reduction in occipital activity, accompanied by a relative increase in frontal activity [25,26], which is discussed as a posterior-anterior shift in aging (PASA) [27]. Further, working memory performance decrements in older adults seem to be associated with structural alterations [28,29] in cerebral microcirculation, which implies age-related decline in microvascular density, thereby contributing to a reduction in cerebral blood flow [30,31]. This, in turn, is likely to reduce metabolic support for neural signaling, especially when levels of neural activity are high, thus contributing to a decline in cognitive performance [30].

One method to investigate the different cortical hemodynamic activation patterns during cognitive testing is functional near-infrared spectroscopy (fNIRS). fNIRS is an optical neuroimaging method that allows for measuring brain tissue concentration changes of oxygenated (O_2_Hb) and deoxygenated hemoglobin (HHb) in the cortex, based on the different absorption spectra of the two chromophores in the near-infrared wavelength range [32]. By shining near-infrared light (650–950 nm) on the scalp and placing a detector a few centimeters apart, changes in the amount of diffuse light reaching the detector provide a measure of changes in cerebral hemoglobin concentrations [33]. Moreover, fNIRS is a non-invasive, safe, portable, and relatively low-cost brain imaging method that is especially well-suited for exercise-cognition research, as it is fairly robust against motion artifacts and has a relatively short preparation time [33,34,35]. Cognitively demanding tasks, such as the n-back task [36], typically lead to an increase of O_2_Hb and an anticorrelated decrease of HHb (i.e., the hemodynamic response function) in the blood because of changes in cerebral blood flow due to neurovascular coupling [37,38]. This is interpreted as an indicator of functional brain activity [39,40,41]. The benefit of applying functional neuroimaging methods, such as fNIRS, in the field of acute exercise-cognition research is that it allows investigating to what extent exercise-induced changes in neural processes underly behavioral outcomes [5].

So far, only three studies have investigated the effects of acute exercise on executive functions in healthy older adults using fNIRS out of which one investigated working memory performance [42] and two inhibitory control [43,44]. In older adults, benefits on working memory performance following an acute bout of light intensity exercise have been found to be accompanied by increased cerebral blood flow in the left PFC while performing a delayed match-to-sample task [42], whereas benefits on inhibitory control (i.e., the Stroop task) have been reported to be accompanied by increased activation bilaterally in the dorsolateral prefrontal cortex (DLPFC), ventrolateral prefrontal cortex (VLPFC) [44], and right frontopolar area [43] following an acute bout of moderate-intensity exercise. This has been interpreted as a compensatory process in light of the HAROLD model [20]. Due to limited evidence from studies with older adults, studies with younger adults might serve to narrow down possible acute exercise effects in older adults. In young adults, benefits on working memory performance following an acute bout of light-intensity exercise were accompanied by increased cerebral blood flow in the right VLPFC at low levels of task demands in a spatial working memory task [45]. Conversely, a supramaximal intensity (i.e., Wingate anaerobic test) showed no beneficial effect on 2-back task performance despite higher prefrontal oxygenation in both hemispheres in young adults [46]. Most fNIRS studies that assessed acute exercise-induced effects in young adults, however, have been conducted in the domain of inhibitory control [47,48,49,50,51]. These studies revealed a beneficial effect of an acute bout of exercise on inhibitory control (i.e., Stroop [47,48,49,50] and Go-/No-Go task performance [51]). This was shown particularly for acute moderate [48,49,51], but also for acute light [43] and high-intensity intermittent exercise [50]. In young adults, these performance increments were accompanied by increased cerebral blood flow either in the left DLPFC [49,50], the left DLPFC and FPA [47], or bilaterally in the DLPFC [48] for the Stroop task. For the Go/No-Go task, decreased cortical activation bilaterally in the DLPFC and in the supplementary motor area was associated with better performance in young adults [51]. Taken together, the beneficial effects of acute exercise seem to be independent of age, but the cortical hemodynamic activation patterns related to improved task performance appear to depend on the executive control task and differ between younger and older adults.

Another influencing factor on the exercise-cognition relationship is the time point of cognitive task assessment post-exercise [5,6]. Overall, studies with both young [44,45,46,47,48,49,50,51] and older adults [42,43,44] either looked at the immediate effects (up to 5 min post-exercise) of acute exercise [44,51] or were restricted to a time window ranging from 5 min [45,46,47,48], 10 min [42], 15 min [43,49,50], up to 30 min [45] post-exercise and investigated inhibitory control [43,44,47,48,49,50,51] or working memory performance [42,45,46]. However, systematic investigations of the persistence of acute exercise effects are missing. As a result, little is known about the development and persistence of acute exercise effects over time after exercise cessation. This, however, can be seen as a prerequisite regarding the practical relevance of acute exercise (e.g., active work breaks) or the design of exercise programs in clinical settings (e.g., stroke rehabilitation).

To sum up, past studies with older adults suggest a positive link between acute moderate-intensity exercise and inhibitory control [43,44], but evidence on exercise-induced changes on working memory remains scarce [42]. Moreover, investigations have been restricted to the immediate effects within a time window of 30 min post-exercise in young adults and 15 min post-exercise in older adults. Also, the underlying cortical hemodynamic activation patterns need further investigation. Therefore, the aim of this study was threefold. First, we aimed to investigate how an acute bout of moderate-intensity exercise (50% VO_2_-peak) affects working memory performance at three time points after exercise cessation (i.e., post 15 min, post 30 min, and post 45 min) in a sample of healthy older adults. Secondly, we applied multichannel fNIRS to examine exercise-induced changes in cortical hemodynamic activation patterns. Thirdly, we assessed how changes on the behavioral level (i.e., 2-back task performance) were related to exercise-induced changes in cerebral blood flow in task-relevant regions, mainly the fronto-parietal working memory network. Based on the current literature on inhibitory control and working memory in young and older adults, we hypothesized that an acute bout of moderate-intensity exercise improves working memory performance, as assessed by the 2-back task and that these performance improvements would persist at least up to 15 min after exercise cessation. With regard to cortical hemodynamic activation patterns, we expected higher cortical activation in frontal, compared to parietal brain regions in both groups. Within frontal brain regions, we expected the experimental group (EG) to show higher cortical activation than the control group (CG) at least up to 15 min post-exercise. Further, we expected the exercise-induced increase in cerebral blood flow in left frontal brain regions to be positively related to 2-back task performance.

## 2. Materials and Methods

### 2.1. Participants

Forty-four healthy, right-handed [52] older adults between 64 and 79 years of age (mean age: 69.18 years ± 3.92; 21 females) participated in this study. Participants were re-recruited as part of a study within the DFG (German Research Foundation, Bonn, Germany) Priority Program SPP 1772 “Multitasking”. Participants were recruited from the participant pool of the Cognition, Brain and Movement Lab of Chemnitz University of Technology (Chemnitz, Germany), newspaper articles and radio announcements by the press office and cross media department of the university, and in person during university lectures for older adults. All participants took part voluntarily in the study and provided written informed consent. Further, they provided medical clearance from a cardiologist to participate in the cardiovascular fitness test (VO_2_-peak).

Participants received no monetary compensation. The study was approved by the local ethics commission of the faculty of Behavioral and Social Sciences of Chemnitz University of Technology, Germany (V-280-17-CVR-Multitasking-29062018) and was conducted in accordance with the latest version of the Declaration of Helsinki. Interested volunteers were screened for the following exclusion criteria in an initial telephone interview: (a) Age range violations (<65 and >80 years; an exception was made for one participant aged 64 as the spouse was already enrolled in the study), (b) use of walking aids, (c) former or current health impairments (severe cardiovascular diseases, such as heart attacks, neurological diseases, stroke; and motor impairments limiting the participant to walk uninterrupted for 30 min, vision impairments or current relevant injuries), (d) obesity (Body Mass Index (BMI) cut-off: BMI > 30), and (e) left-handedness. All interested eligible participants were sent a comprehensive questionnaire concerning demographics, health status, and physical activity level (adopted version of the Baecke Physical Activity Questionnaire) [53], which they were asked to bring with them on their first testing day. Further exclusion tests were applied based on participants’ first laboratory test day: (a) Screening for hand dominance [52], (right handers only), (b) Mini Mental State Examination (MMSE) cut-off: <27 [54], and (c) abnormalities in the Freiburg Visual Acuity Test (FrACT; version 3.9.0) [55]. No participant had to be excluded based on these tests. After screening and exercise testing, participants were matched with respect to age, cardiovascular fitness level, and gender (cf. Table 1) and either assigned to an EG or a CG. Sample size was calculated using G*Power [56]. We assumed a small to moderate effect size of f² = 0.20 (alpha = 0.05, 1 - *p* = 0.80, numerator df = 1, number of groups = 2, number of measurements = 4) based on a previous study by Hyodo and colleagues [43]. A total sample of *N* = 36 was estimated to achieve sufficient statistical power to detect differences between the two groups. Taking attrition of typically 20% into account, we recruited a total sample of *N* = 44 older adults. Data from two participants were excluded from all analyses due to a lack of understanding of the cognitive task. The characteristics of the remaining 42 participants are summarized in Table 1. We had to exclude another seven participants from the fNIRS ANOVA as their fNIRS data were incomplete, either for technical reasons (*n* = 3), insufficient fNIRS signal quality (*n* = 3) at one time-point, or due to physical discomfort of the participant (*n* = 1), resulting in a sample of *n* = 35 for the fNIRS ANOVA (cf. Table S1).

### 2.2. Behavioral Measurements

#### 2.2.1. Working Memory Task

Working memory performance was assessed by use of a letter n-back task. Stimuli consisted of white letters in Arial font with a stimulus object size of 180 pixels, which were presented in the center of a screen on a black background (24 inch monitor (IIYAMA G-Master GB2488HSU-B2, IIYAMA CORPORATION, HA Hoofddorp, The Netherlands, screen resolution of 1920 × 1080 pixels)). The software, Presentation, version 20.3, Build 02.25.19 (NeuroBehavioral Systems, Inc., Berkeley, CA, USA) was used for stimulus presentation and behavioral recordings (reaction time (RT) and accuracy (ACC)). Each stimulus was presented for 500 ms followed by a response interval of 1500 ms in which a white fixation cross was displayed instead of the stimulus, resulting in a total response window of 2000 ms. A randomized stimulus onset jitter between 800–1200 ms (on average 1000 ms) was implemented, resulting in an averaged total trial time of 3000 ms. The participants’ task was to decide whether the displayed letter matched the letter in the previous trial (1-back condition) or two trials before (2-back condition). In the 0-back condition, the letter “X” served as the target, whereas in the 1-back and 2-back condition, the target was randomly selected out of a set of 20 consonants. All conditions were presented randomly in two blocks of 15 trials, of which five were target trials. Every condition was followed by a rest period of 27 s, during the last 5 s of which the instructions for the next trial were displayed. Every block had a duration of 45 s. For each trial, participants indicated whether the stimulus was a target (= match) trial by pressing a green button with their index finger (right arrow key), or a non-target (= non-match) trial by pressing a red button with their middle finger (down arrow key) installed on a German keyboard. Before the experiment started, all participants performed either one or two practice blocks per condition (15–30 trials) to familiarize themselves with the task. The total duration of the n-back task was 6:45 min. Participants were seated comfortably in a quiet, dimly lit room at a viewing distance of approximately 90 cm from the screen. Participants were instructed to respond as fast and as accurately as possible.

#### 2.2.2. Perceived Difficulty of the Working Memory Task

As cognitive load has a crucial impact on behavioral performance, participants were asked to indicate the perceived difficulty of each load condition and the control condition of the n-back task on Borg’s 6–20 RPE scale [57] for each time point.

#### 2.2.3. Behavioral Data Preprocessing

Behavioral data of the n-back task were preprocessed using R 3.6.3 [58] and RStudio 1.2.5033 [59], with speed (RT) and ACC as outcome measures for behavioral performance. The first two trials from every block for each load condition were discarded from analysis, since in the 2-back condition the first two trials are necessarily non-match trials, resulting in 26 trials per condition and time point. In case of a premature response (RT < 200 ms) [60], or a response omission (RT > 2000 ms) [61], a trial was counted as an error, resulting in a response window between 200 ms and 2000 ms after stimulus onset. N-back task performance was included if a participant reached at least 73% correct responses corresponding to 19 out of 26 trials (99% quantile of the binomial distribution with *N* = 26 and *p* = 0.5) in the 1-back condition at pretest (exclusion of *n* = 1 participant). Further, standardized *z*-scores were used to identify and discard extreme values (|*z*| > 3.29) in the rate-correct score (cf. below) across all participants for each combination of load condition and time point (exclusion of *n* = 1 participant). Further, we combined RT and ACC into a single performance measure to control for speed–accuracy trade-offs [62], by use of the rate-correct score (RCS) as an index of response speed adjusted for errors [63]. For statistical analysis, the RCS was computed for each condition for all time points as participants’ number of correct responses divided by the sum of RTs for all responses (both correct and incorrect). The RCS can be interpreted directly as correct responses per time unit (seconds in our case). A higher RCS represents better overall performance on the n-back task.

### 2.3. fNIRS Measurements

Concentration changes in the amount of oxygenated (O_2_Hb), deoxygenated (HHb), and total hemoglobin (HbT) in the cortex were recorded by use of two portable, continuous-wave fNIRS optical tomography systems (NIRSport 88, NIRx Medical Technologies LLC, New York, NY, USA) in a single-subject tandem setup. The montage setup consisted of 16 illuminating sources (LED; time multiplexing) and 16 detectors (silicon photodiode), arranged at an inter-optode distance of approximately 3 cm, providing an adequate compromise between depth sensitivity and signal-to-noise ratio [64]. The NIRScaps for optode placement (EASYCAP GmbH, Herrsching, Germany) were available in four different sizes (head circumferences of 54 cm, 56 cm, 58 cm, and 60 cm) and suitable for all participants. The center of the NIRScap was placed according to the international 10–20 system over the vertex (Cz), by marking the halfway point between nasion and inion and the left and right preauricular points [65]. A retaining overcap (EASYCAP GmbH, Herrsching, Germany) was attached on top of the NIRScap to ensure that no ambient light from other sources (e.g., sunlight, room light) than the fNIRS device could interfere with the fNIRS signal. All data sets were recorded using the acquisition software NIRStar 15.2 (NIRx Medical Technologies LLC, New York, NY, USA) at a sampling rate of 3.47 Hz and two wavelengths of near-infrared light (760 nm for HHb and 850 nm for O_2_Hb). Each recording was preceded by an automatic calibration process, as implemented in the acquisition software NIRStar 15.2 (NIRx Medical Technologies LLC, New York, NY, USA) to determine an optimum amplification factor of 0.4–4.0 V for each channel (i.e., source-detector combination). Participants were asked to avoid head movements, frowning, jaw clenching, or talking during the experiment to minimize extracerebral contamination of the fNIRS signal.

#### 2.3.1. fNIRS Probe Placement and Region of Interest (ROI) Definition

**Figure 1:**
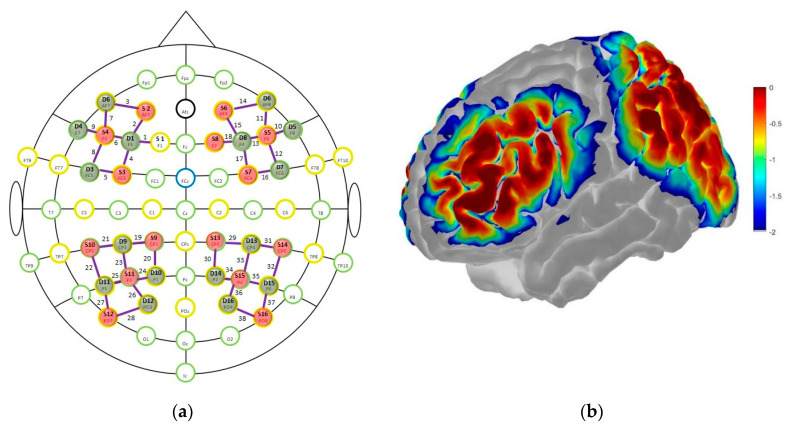
(**a**) For the fNIRS measurements, sources (S) and detectors (D) were positioned according to the international 10–20 system; (**b**) results of the Monte-Carlo simulation based on 1 × 10^-7^ photons (per optode) over the frontal and parietal cortex from a lateral view; the colorbar unit represents the spatial sensitivity of the fNIRS measurements. It is expressed in mm^−1^ and values range from 0.01 to 1 in log10 units: −2 to 0.

We used the automated anatomical labeling atlas Brodman [66], as implemented in the fNIRS Optodes’ Location Decider (fOLD) to assign cortical hemodynamic changes during the 2-back task to specific brain regions [67]. For the frontal cortex, we set the fNIRS probes to cover the left (channel 1–4) and right (channel 14, 15, 17, 18) DLPFC (BA 9/46) and the left (channel 5–9) and right (channel 10–13, 16) VLPFC (BA 44/45). For the parietal cortex, we set the fNIRS probes to cover the left (channel 19, 21–23, 25) and right (channel 29, 31–33, 35) inferior parietal lobe (IPL; BA 39/40) and the left (channel 20, 24) and right (channel 30, 34) superior parietal lobe (SPL; BA 7) (cf. Figure 1a). Based on this assumption, we excluded all channels from further analysis which did not cover our ROI, namely the fronto-parietal network, that were channels 26 (S11-D12), 27 (S12-D11), and 28 (S12-D12) on the left hemisphere, and channels 36 (S15-D16), 37 (S16-D15), and 38 (S16-D16) on the right hemisphere. Overall, this procedure led to a montage setup with 18 channels covering the bilateral DLPFC and VLPFC, and 14 channels covering the bilateral IPL and SPL. An overview of the spatial organization and the sensitivity profile of the fNIRS optode placement is shown in Figure 1. A detailed overview of all channels and their respective international 10–20 system position [68] and corresponding ROI can be found in the supplements (cf. Table S2). The probabilistic path of photon migration through the head for the sensitivity profile was estimated using the Monte-Carlo photon transport software tMCimg via the Atlas Viewer from HomER2 [69,70] (cf. Figure 1b).

#### 2.3.2. fNIRS Data Preprocessing

Preprocessing was performed in Matlab R2018a (The MathWorks, Natick, MA, USA) and by use of additional scripts from the HomER2 toolbox, version 2.3 [69]. First, the raw optical intensity time series data were converted to changes in optical density (OD) using the hmrIntensity2OD function [71]. We plotted the power spectrum of every O_2_Hb time series to assess the quality of the fNIRS signal. Since a frequency peak of the cardiac activity around 1 Hz in the O_2_Hb signal indicates good contact between the optical probe and the scalp [72], every channel that did not match this criterion was excluded from further analysis. Following this procedure, 11.43% of the data across all participants and time points were removed from further analysis. Correction for motion artifacts was performed using wavelet filtering, which has been described as a promising approach to reduce the influence of motion artifacts [71]. We used an algorithm described by Molavi and Dumont [73], as implemented in the HomER2 hmrMotionCorrectWavelet filtering function. The algorithm applies a probability threshold for removing outlying wavelet coefficients, which were assumed to correspond to motion artifacts. We used a threshold of 0.1 times the inter-quartile range, as recommended [73]. We then applied a band-pass filter (third-order low-pass and fifth-order high-pass Butterworth filter) with cut-off frequencies of 0.01–0.08 Hz to remove physiological noise like cardiac frequency, respiratory frequency, Mayer waves, and very low-frequency oscillations [39]. Then, the OD time-series data were converted into concentration changes expressed in units of molar × 10^−8^ of O_2_Hb, HHb, and HbT using the modified Beer-Lambert law [74], that included an age-dependent differential path length factor (DPF) using the following formula: DPF_807_ = 4.99 + 0.067 × A^0.814^, where DPF is the DPF measured at 807 nm and A is age in years [75]. All trials related to the same condition and time point were block averaged (time window: −2 to 45 s) using the HomER2 hmrBlockAvg function to recover the mean hemodynamic response. Finally, to remove remaining outliers, we standardized the range of each condition for each time point across all channels by use of standardized *z*-scores and excluded all channels outside the range of |3.29| (99.95% quantile of the standard normal distribution). Based on this approach, 1.58% of the data were removed from further analysis. For statistical analysis, we combined O_2_Hb and HHb into a single measure of cortical activation by use of hemoglobin difference (HBdiff = O_2_Hb − HHb) which represents oxygen supply (saturation as measured by O_2_Hb) versus demand (extraction as measured by HHb) [76].

### 2.4. Cardiovascular Fitness Test

Cardiovascular fitness was assessed by spiroergometry (ZAN600 CPET, nSpire Health, Oberthulba, Germany) on a stationary bicycle (Lode Corival cpet, Groningen, The Netherlands) by use of a ramp protocol to determine participants’ peak oxygen consumption (VO_2_-peak). A ramp protocol with a progressively increasing load of 15 W/min, starting with 10 W, was used for women and a progressively increasing load of 20 W/min, starting with 20 W, was used for men. Participants were told to keep their revolutions per minute (rpm) between 60 and 80. All tests were supervised by an experienced sports scientist. Electrocardiography (recorded with a twelve-lead ECG fully digital stress system; Kiss, GE Healthcare, Munich, Germany), breath-by-breath respiration, heart rate, and blood pressure were monitored continuously. Every two minutes, participants were asked to indicate their rate of perceived exertion (RPE) on Borg’s 6–20 RPE scale [57]. The scale ranged from 6 = “very easy” to 20 = “extremely difficult”.

All spiroergometry protocols started with a 3 min rest period and finished with a 5 min cool-down period (1 min initial load and then no load). Tests were terminated due to volitional exhaustion, or (at the latest) by reaching a respiratory exchange ratio of ≥1.05–1.10 for more than 30 s. Further, protocols were terminated if the maximum age-predicted heart rate was reached (approximately >(220–age)) or a risk factor occurred (i.e., systolic blood pressure ≥230/115 mmHg, abnormal ECG response). Participants’ VO_2_-peak was determined by the average of the last five values of the last fully completed load level (approximately 10 s). The watt level corresponding to 50% of the individual participants’ VO_2_-peak was used to determine the individual intensity of the acute exercise intervention. We opted against a maximal graded exercise test, since raising the exercise intensity to the level of VO_2_-max was considered unsafe for older adults due to medical concerns. Participants were asked to avoid any vigorous exercise and consumption of caffeine and alcohol for at least 12 h before exercise testing.

### 2.5. Exercise Intervention

All participants assigned to the EG cycled at 50% of their individual VO_2_-peak for a duration of 15 min on a stationary bicycle (Lode Corival cpet, Groningen, the Netherlands) with a cadence between 60 and 80 rpm. Participants were fitted with a Polar A300 heart rate (HR) monitor (Polar Electro Oy, Kempele, Finland) with an H7 HR sensor (Polar Electro Oy, Kempele, Finland) to measure their HR during the exercise intervention, just before commencement (pre) and during the n-back task at multiple time points post-exercise (i.e., post 15 min, post 30 min, and post 45 min). Further, participants were asked to indicate their rate of perceived exertion (RPE) by pointing with their index finger on Borg’s 6–20 RPE scale [57], just before the start, every 2 min during, and at the end of the exercise intervention as an indicator of physiological arousal and to control for exercise intensity. 

We chose a moderate intensity corresponding to 50% of participants’ VO_2_-peak and lasting for 15 min, since the largest benefits for executive control tasks have been associated with moderate-intensity exercise and a duration of 10–20 min [6,77]. Additionally, cerebral blood flow has been reported to increase between low and moderate exercise intensities, to remain stable from moderate-to-hard intensities and then to decline at very hard or maximal intensities to oxygenation values similar to those observed during low-intensity exercise [78].

**Figure 2:**
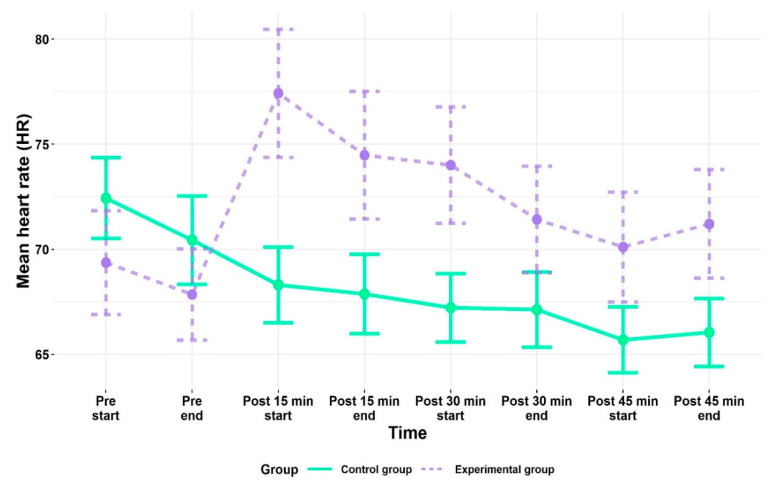
Mean heart rate (HR) values for the experimental (EG) and control group (CG) before (pre) and post-exercise (follow-up measurements) for the beginning (start) and the end of each n-back task. All values are given as mean (*M*); error bars represent one standard error (*SE*) of the mean.

The watt level during the exercise intervention ranged from 40 W to 104 W, with a mean of 68.11 W ± 15.23 (cf. Table 1 for VO_2_-peak values). At the end of the acute exercise intervention, average heart rate and RPE were 119.84 ± 17.90 beats per minute (bpm) (cf. Figure 2) and 13.63 ± 2.14 points on Borg’s 6–20 RPE scale [57], respectively, which corresponds to moderate-intensity exercise according to the guidelines of the American College of Sports Medicine [79]. At the start of the first n-back task after the exercise intervention (i.e., post 15 min), participants’ HR was, on average, 77.42 bpm (*SD* = 13.26), that is 11.57% higher (*SD* = 9.08) than their average HR at rest. After 45 min, the averaged HR was 70.11 bpm (*SD* = 11.43), that is 1.28% higher (*SD* = 8.53) than the average HR at rest; thus, HR values returned to baseline approximately at the start of the last follow-up measurement (i.e., post 45 min).

As expected, the HR values of the CG remained relatively stable from time point pre to post 45 min. All HR values before (pre) and post-exercise (follow-up measurements) for the beginning (start) and the end of each n-back task can be found in the Supplementary Materials (cf. Table S3).

### 2.6. Design and Testing Procedures

**Figure 3:**
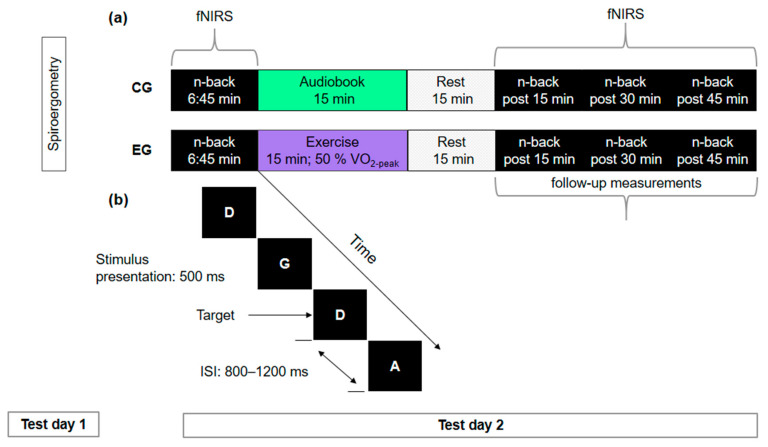
(**a**) Schematic illustration of the design and testing procedures consisting of two test days for the experimental (EG) and control group (CG). Using functional near-infrared spectroscopy (fNIRS), cortical hemodynamic activation was measured while participants performed the letter n-back task. (**b**) Schematic illustration of the 2-back condition of the n-back task. Stimulus presentation time was 500 ms, inter stimulus interval (ISI) was 800–1200 ms (on average 1000 ms). Participants were allowed to respond up to 2000 ms.

This study was designed as a between-subjects pre- and post-test comparison. Participants visited the laboratory twice. On their first visit, they returned the questionnaires concerning demographics, health status, and physical activity level which they had filled out at home. In addition, all participants performed the cardiovascular fitness test (spiroergometry). During their second visit, all participants assigned to the EG performed the n-back task (cf. Figure 3b) before exercising at 50% of their VO_2_-peak for 15 min on a stationary bicycle (Lode Corival cpet, Groningen, The Netherlands), as well as 15 min, 30 min, and 45 min after exercise cessation (cf. Figure 3a). Participants in the CG listened to an audiobook for 15 min instead of exercising. fNIRS data were recorded before exercise and post 15 min, post 30 min, and post 45 min, but not during exercise or while listening to the audiobook in the control condition, respectively.

### 2.7. Statistical Analysis

All statistical analyses were performed using R 3.6.3 [58] and RStudio 1.2.5033 [59]. The additional packages “ez” [80] and “emmeans” [81], were used for the mixed repeated measures analysis of variance (ANOVA) and post-hoc comparisons. Plots were created with the “ggplot2” package [82]. Perceived difficulty of the n-back task was analyzed by a 2 (group: Experimental, control) × 4 (time: Pre, post 15 min, post 30 min, post 45 min) × 3 (load condition: 0-back, 1-back, 2-back) mixed repeated measures ANOVA, with group as the between-subjects factor and time and load condition as within-subjects factors. Due to ceiling effects in the control condition (0-back) and in the low-working memory load condition (1-back) in terms of ACC and as cognitive load has a crucial impact both on behavioral performance and neural effort, we limited our analysis to the 2-back condition.

Behavioral data (RCS) were analyzed with a 2 (group: Experimental, control) × 4 (time: Pre, post 15 min, post 30 min, post 45 min) mixed repeated measures ANOVA with group as between-subjects factor and time as within-subjects factor under the highest cognitive load condition (i.e., 2-back). Further, to test our hypothesis that acute exercise effects would persist at least up to 15 min after exercise cessation and to assess performance improvements over the course of the experiment within each group, we used planned contrasts to calculate differences between time point pre and the three follow-up measurement time points for each group. 

For the hemodynamic data, HBdiff for the DLPFC and VLPFC were strongly correlated (*r* = 0.78, *p* < 0.001 for left hemisphere; *r* = 0.83, *p* < 0.001 for right hemisphere) and did not differ significantly. Likewise, the IPL and SPL data were strongly correlated (*r* = 0.92, *p* < 0.001 for left hemisphere; *r* = 0.87, *p* < 0.001 for right hemisphere). Further, HBdiff for the averaged right and left DLPFC and VLPFC data (*r* = 0.81, *p* < 0.001 for frontal cortex), as well as the averaged right and left IPL and SPL data (*r* = 0.91, *p* < 0.001 for parietal cortex) were strongly correlated, as well as the frontal and parietal data within each hemisphere (*r* = 0.73, *p* < 0.001 for left hemisphere and *r* = 0.84, *p* < 0.001 for right hemisphere). We therefore pooled and averaged the DLPFC and VLPFC to “frontal” and the IPL and SPL data to “parietal” by hemisphere. Likewise, the frontal and parietal data were pooled and averaged by hemisphere to the “left” and “right” hemispheres, respectively. fNIRS data (HBdiff) were analyzed with a 2 (group: Experimental, control) × 4 (time: Pre, post 15 min, post 30 min, post 45 min) × 2 (region: Frontal, parietal) × 2 (hemisphere: Left, right) repeated measures ANOVA with group as between-subjects factor and time, region, and hemisphere as within-subjects factors under the highest cognitive load condition (i.e., 2-back). Further, to test our hypothesis that HBdiff values were higher in ROI frontal left compared to the parietal brain regions, we calculated planned contrasts. Additionally, planned contrasts were calculated similarly to the behavioral data, for time point pre compared to the three follow-up measurement time points for each group.

All planned contrasts were corrected according to the Bonferroni procedure, if necessary. If sphericity was violated (using Mauchly’s test), the data were Greenhouse-Geisser-corrected. For all analyses, *p*-values < 0.05 were regarded as significant. Effect sizes were calculated for significant results by generalized eta squared (*η_ges_*^2^). Post-hoc testing was carried out using Welch’s t-test for pairwise comparisons of estimated marginal means (emmeans) with Bonferroni-adjusted alpha levels to determine pre- to post-measurement changes. EG and CG were statistically similar to one another on measures of age, gender, BMI, years of formal education, MMSE, and cardiovascular fitness level, as determined by paired t-tests and a chi-square test for gender (cf. Table 1). Consequently, we abstained from including covariates.

To assess the relation between behavioral and neurophysiological data, correlation and multiple regression analyses via the forced entry procedure were computed for each time point with group and HBdiff for each ROI (frontal left and right, parietal left and right) as predictors for behavioral performance (RCS) under the highest cognitive load condition (i.e., 2-back) in the regression models. We abstained from including BMI in the correlational analysis, as all participants had normal weight. From the remaining factors, only the MMSE revealed a significant correlation coefficient, which was then included into the regression models.

## 3. Results

### 3.1. Behavioral Results—Rate-Correct Score (RCS)

**Figure 4:**
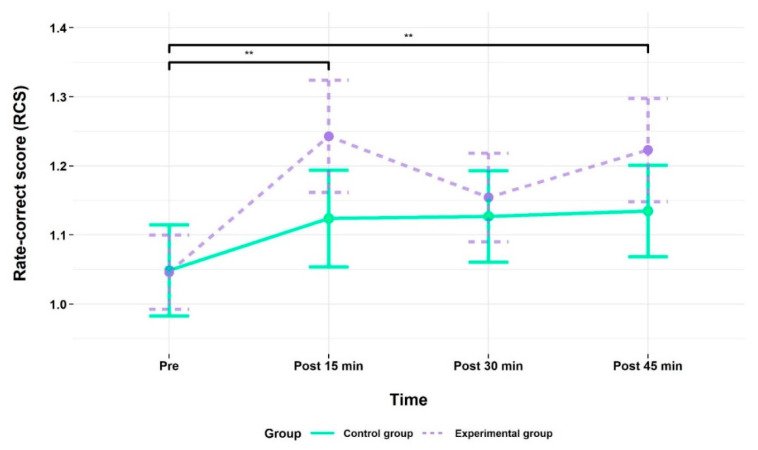
2-back task performance as measured by the rate-correct score (RCS) for the experimental (EG) and control group (CG) at time point pre (baseline) and at the follow-up measurements after the acute exercise intervention (EG) or listening to the audiobook (CG), respectively. All values are given as mean (*M*); error bars represent one standard error (*SE*) of the mean. ** *p* < 0.01.

As shown in Figure 4, working memory performance improved in both groups from time point pre to post 15 min at which performance differences between the two groups were most pronounced in favor of the EG. This is evidenced by a higher RCS, indicating better overall performance on the 2-back task. For RCS data, the repeated-measures ANOVA revealed a main effect of time, *F* (2.75, 110.05) = 6.54, *p* = 0.001, *η*_ges_^2^ = 0.03, indicating that performance improved over time.

For time, a priori planned contrasts revealed for the EG a significant difference in 2-back task performance between time points pre (*M*: 1.05, *SE*: 0.05) and post 15 min (*M*: 1.24, *SE*: 0.08; *p* ≤ 0.001), and also post 45 min (*M*: 1.22, *SE*: 0.07; *p* = 0.001), indicating significant performance improvements with reference to the time point pre. For the CG, no such differences were found in any contrast. All behavioral results (RCS, perceived difficulty) and the values that were used to calculate the RCS (RT, ACC) are summarized in Table 2. The corresponding values for these parameters for the control condition (0-back) and the low-working memory load condition (1-back) can be found in the Supplementary Materials (cf. Tables S4 and S5).

### 3.2. Behavioral Results—Perceived Diffifulty

**Figure 5:**
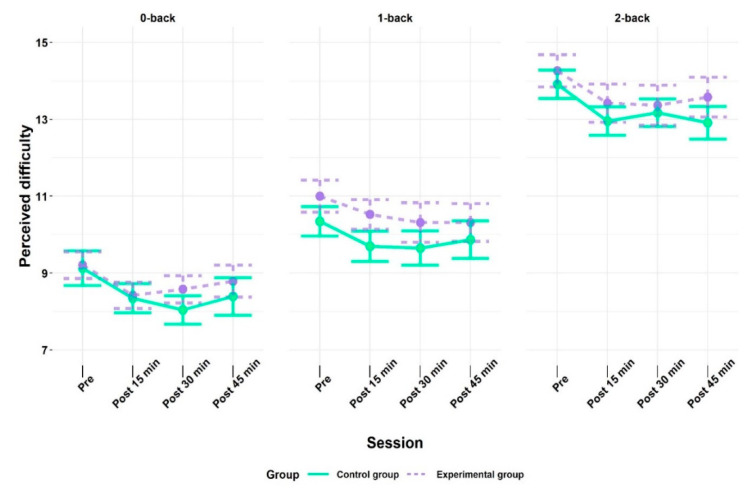
Perceived difficulty of the n-back task indicated by the experimental (EG) and control group (CG) at time point pre (baseline) and at the follow-up measurements after the acute exercise intervention (EG) or listening to the audiobook (CG), respectively. All values are given as mean (*M*); error bars represent one standard error (*SE*) of the mean.

Perceived difficulty of the n-back task increased as a function of cognitive load from the control condition (0-back) up to the high working memory load condition (2-back) in both groups (cf. Figure 5). This was confirmed by a main effect of condition, *F* (1.54, 61.48) = 187.45, *p* < 0.001, *η_ges_*^2^ = 0.53. Further, the main effect of time, *F* (2.17, 86.81) = 6.64, *p* = 0.002, *η_ges_*^2^ = 0.02, was statistically significant, indicating that perceived difficulty declined over time. All interaction effects were non-significant (cf. Table S6). Post-hoc analyses indicated that perceived difficulty increased as a function of condition (i.e., cognitive load) from 0-back (*M*: 8.55, *SE*: 0.13) to 1 back (*M*: 10.15, *SE*: 0.14), as well as from 0-back to 2-back (*M*: 13.41, *SE*: 0.14) and from 1-back to 2-back. Further, the “pre” time point (*M*: 11.29, *SE*: 0.24) was perceived as significantly more difficult compared to all other time points: post 15 min (*M*: 10.54, *SE*: 0.24), post 30 min (*M*: 10.50, *SE*: 0.25), and post 45 min (*M*: 10.62, *SE*: 0.26). However, none of the other time points varied in perceived difficulty.

### 3.3. fNIRS Results

**Figure 6:**
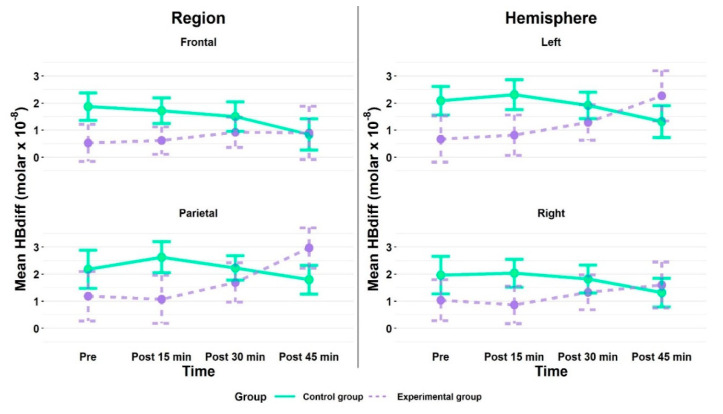
Mean cortical hemodynamic activity as measured by HBdiff in region (frontal, parietal) and hemisphere (**left**, **right**) during the 2-back task for the experimental (EG) and control group (CG) at time point pre (baseline) and at the follow-up measurements after the acute exercise intervention (EG) or listening to the audiobook (CG), respectively. All values are given as mean (*M*); error bars represent one standard error (*SE*) of the mean.

Overall, mean cortical hemodynamic activation, as measured by HBdiff, was higher in the parietal than the frontal brain regions in both groups and was similar for both hemispheres. When comparing the activation between both groups, the CG showed higher values in both regions (frontal, parietal) and hemispheres (left, right) at almost all time points (cf. Figure 6 and Table S7). The ANOVA confirmed a main effect of region, *F* (1, 33) = 4.59, *p* = 0.040, *η_ges_*^2^ = 0.012, with higher values for the parietal (*M*: 1.99, *SE*: 0.24) than frontal (*M*: 1.14, *SE*: 0.21) brain regions. All other main and interaction effects yielded non-significant results (cf. Table S8). The planned contrasts between the ROI frontal left and parietal brain regions for each group, as well as between time point pre and the three follow-up measurement time points for each group were non-significant (all *p* > 0.05).

**Figure 7:**
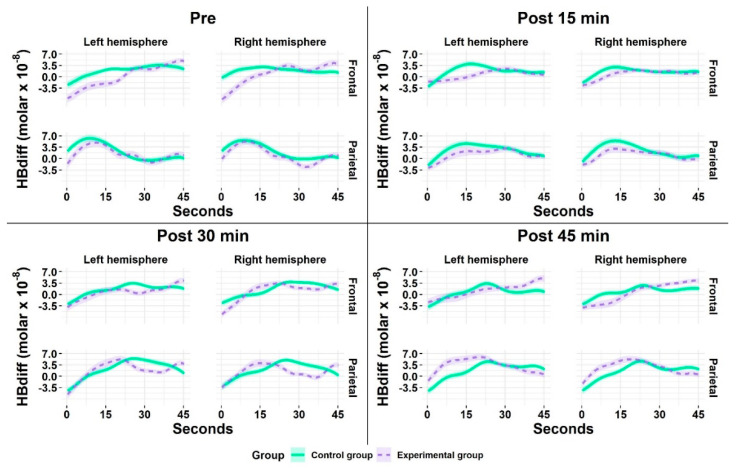
Mean cortical hemodynamic activity as measured by HBdiff during the 2-back task for the experimental (EG) and control group (CG) at the time point pre (baseline) and at the follow-up measurements after the acute exercise intervention (EG) or listening to the audiobook (CG), respectively. All values are given as mean (*M*); the colored frames on the curves correspond to one standard error (*SE*) of the mean.

Figure 7 reveals the cortical activation patterns across the 45 s measurement time of the 2-back task. Both groups demonstrated an almost similar time course of activation (i.e., initial increase followed by a peak with a subsequent decrease) with nearly identical magnitude of activation while performing the 2-back task.

### 3.4. Interrelation of HBdiff and Behavioral Performance

We performed correlation and regression analyses to further examine the relationship between HBdiff and 2-back task performance over time (cf. Table 3).

There was a significant correlation between the participants’ MMSE score and RCS performance at time point pre only (*r* = 0.20, *p* < 0.001). No significant association was found between RCS performance and gender, age, education, and VO_2_-peak. Thus, we abstained from including these variables in the regression models. Overall, the regression model with group, HBdiff for each ROI (frontal left and right, parietal left and right), and MMSE as predictors for RCS performance was significant for time point pre (*p* = 0.023). The ROI frontal left significantly predicted RCS performance at time point post 15 min (*B* = 0.08, *p* = 0.013). Hence, if HBdiff in the frontal left ROI increases by 1 (expressed in units of molar × 10^−8^ of HBdiff), the RCS is estimated to increase by 0.08. All other predictors did not significantly explain variance (cf. Table 3).

## 4. Discussion

The present study was designed to examine the effects of a 15 min acute bout of moderate-intensity exercise on working memory performance. In order to advance knowledge about acute exercise effects from a neuro-cognitive perspective, cortical hemodynamic activity was assessed during cognitive testing before and at three time points post-exercise (i.e., post 15 min, post 30 min, and post 45 min). We found a significant time effect for working memory performance, but no interaction with the group. Yet, within the exercise group, 2-back task performance was significantly enhanced 15 min and 45 min post-exercise, indicating that exercise might have at least slight effects on working memory performance. Regardless of group, higher cortical activation in the working memory core network (i.e., left frontal) was associated with higher working memory performance at time point post 15 min.

### 4.1. Effects of Moderate-Intensity Exercise on Behavioral Performance

When interpreting the main effects, behavioral performance analysis did not point to a significant influence of group allocation or measurement time point on 2-back task performance, as measured by the RCS. Thus, we need to assume that the acute exercise intervention did not enhance executive performance compared to the control condition (i.e., listening to an audiobook). However, on the descriptive level and on the level of a priori planned contrasts, the behavioral data suggest an improvement of 2-back task performance due to an acute bout of moderate-intensity exercise, especially 15 min and 45 min post-exercise, thereby providing slight evidence for the beneficial effects of moderate-intensity exercise on working memory performance in older adults. This is in line with previous studies which investigated the effects of acute moderate-intensity exercise on working memory [42,45] and inhibitory control [43,49], by use of a comparable exercise protocol or high-intensity intermittent exercise [50]. However, due to missing interaction effects, the results need to be interpreted with caution.

Despite task familiarization prior to the start of the experiment, practice effects in both groups may have occurred. This is supported by the higher subjective difficulty rating of the first 2-back task run compared to all follow-up measurement time points. One explanation for this might lie in successful strategy use [83] and a less demanding stimulus-response relation due to more automatic motor responses, namely the process of rule learning over time [84]. One option to lower the influence of practice on task performance might have lain in increasing task demands. However, we abstained from including a 3-back condition in our final experimental design as our pilot study had resulted in participants partly performing at chance level.

When investigating the development and persistence of acute moderate-intensity exercise over time, lower intensities have been shown to benefit cognitive performance immediately after exercise, whereas higher exercise intensities have been associated with benefits in cognitive performance with a delay [6]. Therefore, we assumed moderate-intensity exercise (i.e., 50% of participants VO_2-_peak) to induce improvements in working memory performance at least up to 15 min post-exercise, as has been shown for inhibitory control tasks in young [49] and older adults [43]. This hypothesis was partly confirmed by planned contrasts within the EG, but not by group differences 15 min post-exercise. One explanation might be that the intensity we used was not strenuous enough to induce more distinct cognitive improvements 15 min post-exercise and beyond. On the other hand, studies with young adults comparing the effects of different exercise intensities either during [85,86], or following an acute bout of exercise [48] point to impairments in cognitive functioning when the physical load becomes too heavy, especially in lower fit individuals [86].

Interestingly, studies investigating moderate-intensity exercise effects on inhibitory control in older adults found significant effects [43,44,49,51]. These divergent findings for inhibitory control and working memory suggest that although inhibition and working memory have a close interrelation and share neural networks [87], acute moderate-intensity exercise might exert differential effects depending on the degree of involvement of each executive function in the task. Further, studies reported only weak to moderate correlations between Stroop and 2-back task performance [88,89,90]. Compared to the Stroop task [91], as a classical measure of inhibitory control, the letter n-back task is more vulnerable to aging effects due to its manifold cognitive processing efforts [83,92]. Especially in older adults, mainly attentional and verbal memory capacities seem to play a crucial role in the n-back task [92]. In comparison to the n-back task, we understand the Stroop task as a rather pure inhibitory control task, since color-word reading per se is a more automatic process that has to be voluntarily suppressed by ink color naming. Thus, even though the naming condition of the Stroop task is of higher attentional demand and in particular need of control, it might be less cognitively demanding than the 2-back task. In this context, the unique components of inhibition and working memory might need to be disentangled to further our understanding of why acute exercise might exert differential effects on different executive functions.

Even though meta-analytic evidence generally supports the positive effect of acute exercise across several aspects of cognition [6,7,93,94], one may keep in mind that only a few cognitive functions have been studied in the context of exercise-cognition research (predominantly inhibition) and that mainly young adults have been studied [5]. Our results support the assumption that moderate acute exercise does not necessarily improve cognitive performance [5,6]. It is most likely that rather diverse moderators, such as exercise intensity, duration, modality, the cognitive task applied, and the time point of the post-exercise assessment can potentially influence the extent to which acute exercise may have an enhancing effect on cognition [5,6]. Whether the slight effects of acute moderate intensity exercise on working memory performance in our study are due to exercise intensity, duration, or the cognitive task applied remains speculative.

### 4.2. Effects of Acute Moderate-Intensity Exercise on Cortical Hemodynamic Activation Patterns and Its Relation to Behavioral Performance

This is the first study which investigated the influence of an acute bout of moderate-intensity exercise on working memory performance before and at three time points after exercise cessation (i.e., post 15 min, post 30 min, and post 45 min) in a sample of healthy older adults. Furthermore, we addressed the underlying neural mechanisms by measuring cortical hemodynamic activation patterns using fNIRS. Brain activity was registered in the frontal (DLPFC/VLPFC) and parietal (IPL/SPL) brain regions, while participants performed a letter n-back task to assess the development and persistence of acute moderate-intensity exercise effects over time. Additionally, the relationship between cortical hemodynamic activation patterns and working memory performance was investigated. Overall, we expected cortical hemodynamic activation to be higher in frontal, as compared to parietal brain regions, as described by the PASA model [27] for both groups. Further, we expected cortical hemodynamic activity to be even more pronounced in the EG as a result of an acute exercise-induced increase in cerebral blood flow. Drawing on the results of acute moderate-intensity exercise on working memory [42] and inhibition in older adults [43,44], we expected the EG to show even higher HBdiff values in frontal brain regions compared to the CG at least up to 15 min post-exercise.

Even though there is still some doubt as to whether the recruitment of additional neural resources from anterior regions (e.g., PFC) contributes to the maintenance of cognitive performance in older adults [10,27,95], one potential compensatory mechanism is higher prefrontal activation through upregulation (i.e., an age-related increase in brain activity directly correlated with better performance) [89]. A related hypothesis which might explain the phenomenon of higher cortical activation in frontal, compared to parietal brain regions in older adults is that increases in PFC activity rather reflect reduced neural efficiency or specificity as a result of age-related structural and neurochemical changes [96,97,98,99]. From this point of view, enhanced frontal brain activity does not necessarily contribute to the maintenance of working memory performance in older adults. Even though it is difficult to adjudicate between explanatory approaches based on average activity levels within brain regions, recent evidence suggests that increased PFC activation accompanied by high cognitive task performance reflects compensatory mechanisms. In contrast, increased PFC activation accompanied by lower task performance reflects reduced efficiency or specificity, rather than compensation [90,100,101].

Contrary to our expectations, overall, our results revealed higher cortical activation as measured by HBdiff in parietal, compared to frontal brain regions. Thus, our data do not point to the proposed age-related shift of activation from parietal to frontal brain regions, as supposed by the PASA model [10,27]. Given that parietal activation is related to the storage component of working memory [23] and cortical activation positively relates to the amount of stored items [18], our findings might show that the 2-back task relied more than expected on the storage components of working memory, leading to higher cortical activation in the parietal cortex that superimposed the typical pattern described by the PASA model. To assess how cortical hemodynamic activation patterns are linked to working memory performance over time, we ran a multiple regression analysis for each time point. Our results yielded no significant correlation between the RCS and HBdiff in any ROI at baseline and the follow-up measurements at post 30 min and post 45 min for both groups. Interestingly, the regression model for the time point post 15 min showed a positive effect of left-lateralized brain activation, whereas right-lateralized brain activation was negatively related to RCS performance. This negative effect of right frontal activation, accompanied by a positive effect of left frontal activation, which is related to increased cognitive performance, might suggest that acute exercise does not facilitate compensational reorganization, as assumed by the HAROLD model [20]. Rather, this points to enhanced processing within the working memory core network (i.e., left frontal). This, in turn, is in line with the assumption that compensatory neural networks are less efficient than the original task networks, and thus, in order to establish long-lasting beneficial effects (e.g., more efficient processing), processing within the core network should be maintained or reinforced [95].

Therefore, our results are in contrast to a comparable study by Hyodo and colleagues [43], who report greater bilateral activation, that is, broader activation in line with the HAROLD model [20], during a Stroop task following an acute bout of moderate-intensity exercise. Additionally, planned contrast within the EG between the pre-test and time point post 15 min showed improved RCS performance. Thus, one might speculate that acute moderate-intensity exercise affects the executive processing and attention component of working memory in the frontal cortex [22], to a greater extent than the storage component located in the parietal cortex [18,23,24].

## 5. Limitations

The human aging process is characterized by highly individual trajectories in brain structure and function [102]. Age-related cerebral atrophy (e.g., structural shrinkage) is discussed as one factor that diminishes the sensitivity of the fNIRS measurement. Therefore, the distance between the cortex and the surface of the scalp (where the fNIRS sources and detectors are positioned) increases and measured activation may underestimate the actual cortex activation [103,104]. However, all participants of our sample reported good health with no former or current history of neurological diseases, indicating at least no pathological changes. Yet, by integrating our study results into previous, comparable studies, explanatory power remains a general problem of studies with older adults. Although we used a high-density fNIRS setup by use of a single-subject tandem setup covering frontal and parietal brain regions, the fNIRS montage set-up did not include short separation channels. Instead, all sources and detectors were arranged at an inter-optode distance of approximately 3 cm. Thus, the fNIRS signal might be biased, due to acute exercise evoked systemic changes (e.g., skin blood flow, respiration, blood pressure, sympathetic nervous system activity) in the extracerebral compartment, which might lead to false-positive results [102]. However, we started the post-exercise fNIRS measurements 15 min after exercise cessation and cerebral artery mean blood flow velocities and skin blood flow have been reported to return to values similar to those observed during rest within 15 min after acute moderate intensity exercise [43,49]. Thus, we are confident in assuming that changes in systemic physiology did not influence our results.

## 6. Outlook

Future studies will have to systematically address the extent to which the after-effects of acute exercise depend on moderators such as exercise intensity, duration and modality, the cognitive task applied, and its time point of assessment post-exercise [5,6] to examine the (design) characteristics that facilitate the enhancing effect of acute exercise on cognition. With regard to cortical hemodynamic activation patterns underlying behavioral outcomes, our findings by use of an fNIRS measurement set-up covering both frontal and parietal brain regions highlight the need for whole-head measurement set-ups. The conclusions which can be drawn from the widely used set-ups restricted to the PFC are necessarily limited due to the involvement of frontal and parietal brain regions in executive control tasks. Therefore, further studies in the field of exercise-cognition research are needed, which focus on age-related changes in whole brain cortical hemodynamic activation patterns following different exercise-intensities (moderate vs. high-intensity) and exercise protocols (continuous vs. interval) and executive control tasks. Further, as indicated by the considerable variability in our behavioral and fNIRS data, future research will have to investigate inter-individual differences in response to acute exercise. Ultimately, the human cognitive aging process is characterized by high inter- and intra-individual variability leading to substantial variability within and between participants.

## 7. Conclusions

This study investigated the effects of an acute bout of moderate-intensity exercise (15 min; 50% VO_2_-peak) on working memory performance (i.e., n-back task) in a sample of healthy older adults compared to a resting control condition (i.e., listening to an audiobook). Additionally, we addressed the development and persistence of acute exercise effects and the underlying neural mechanisms by measuring cortical hemodynamic activation patterns using fNIRS during cognitive testing before and at multiple follow-up measurement time points (i.e., post 15 min, post 30 min, and post 45 min). Only within the exercise group, 2-back task performance was significantly enhanced 15 min and 45 min post-exercise, indicating that exercise might have at least slight effects on working memory performance and that effects last up to 45 min. Regardless of group, higher cortical activation in the working memory core network (i.e., left frontal) was associated with better overall n-back task performance at the time point post 15 min. Results support the practical relevance of short active breaks to enhance cognitive performance and might be also transferred to clinical settings in future studies.

## Figures and Tables

**Table 1:** Participant characteristics of the experimental (EG) and control group (CG).

Variable	EG (*n* = 19)	CG (*n* = 23)	*p*
Age (range)	68.26 ± 3.31 (65 − 79)	69.7 ± 4.23 (64 − 79)	0.226
Gender (f|m)	10|9	10|13	0.779
BMI (kg/m^2^)	24.47 ± 2.23	24.91 ± 2.39	0.539
Education (years)	15.66 ± 2.36	15.85 ± 2.31	0.795
MMSE (sum score)	29.28 ± 0.11	29.09 ± 1.16	0.568
VO_2_-peak (mL/kg/min)	25.48 ± 5.98	24.52 ± 6.29	0.617
Heat rate (bpm) at VO_2_-peak	138.55 ± 18.27	135.40 ± 16.94	0.569
Watt (W) at VO_2_-peak	135.84 ± 30.29	139.09 ± 39.61	0.767
Borg score (RPE) at VO_2_-peak	12.30 ± 1.29	12.39 ± 1.83	0.862

Note. All values are given as mean (*M*) ± standard deviation (*SD*). Age = age in years; Gender = female|male, BMI = Body Mass Index expressed in kg/m^2^; Education = years of formal education; MMSE = sum score of the Mini Mental State Examination; VO_2_-peak = peak oxygen uptake during the cardiovascular fitness test expressed in mL/kg/min; bpm = beats per minute; RPE = rate of perceived exertion according to Borg’s 6–20 RPE scale.

**Table 2:** Time course of 2-back task performance for the experimental (EG) and control group (CG).

	Time Point	EG (*n* = 19)	CG (*n* = 23)
		2-back	2-back
RT per trial (ms)	Pre	837.34 ± 37.65	885.07 ± 57.39
	Post 15 min	788.38 ± 47.22	857.52 ± 57.18
	Post 30 min	809.00 ± 43.07	847.58 ± 49.49
	Post 45 min	796.57 ± 46.10	846.37 ± 51.21
ACC (%)	Pre	84.62 ± 2.39	86.62 ± 2.56
	Post 15 min	91.70 ± 1.84	88.46 ± 1.98
	Post 30 min	88.87 ± 1.74	88.80 ± 1.23
	Post 45 min	91.70 ± 1.67	89.30 ± 1.69
RCS	Pre	1.05 ± 0.05	1.05 ± 0.07
	Post 15 min	1.24 ± 0.08	1.12 ± 0.07
	Post 30 min	1.15 ± 0.06	1.13 ± 0.07
	Post 45 min	1.22 ± 0.07	1.13 ± 0.07
Perceived difficulty	Pre	14.26 ± 0.42	13.91 ± 0.37
	Post 15 min	13.42 ± 0.50	12.96 ± 0.37
	Post 30 min	13.37 ± 0.52	13.17 ± 0.36
	Post 45 min	13.58 ± 0.51	12.91 ± 0.43

Note. All values are given as mean (*M*) ± one standard error (*SE*) of the mean; RT = reaction time; ACC = accuracy; RCS = rate–correct score.

**Table 3:** Results of the multiple regression analysis with rate-correct score (RCS) as criterion and group, HBdiff for each ROI (frontal left and right, parietal left and right), and MMSE as predictors for each time point.

		Regression Coefficients				F Statistic			
Time Point	Effect	*B*	*ß*	*T*	*p*	*F*	*df*	*p*	*adjR* ^2^
Pre	Group: EG	0.00	0.00	−0.02	0.986	2.93	6,30	0.023	0.24
	MMSE	0.10	0.38	2.53	0.017				
	Frontal (left)	0.04	0.50	1.51	0.141				
	Frontal (right)	−0.05	−0.74	−1.88	0.069				
	Parietal (left)	0.04	0.60	1.28	0.211				
	Parietal (right)	0.00	−0.08	−0.18	0.857				
Post 15 min	Group: EG	0.18	0.26	1.62	0.116	2.10	6,29	0.083	0.16
	MMSE	0.10	0.31	1.70	0.100				
	Frontal (left)	0.08	0.65	2.64	0.013				
	Frontal (right)	−0.08	−0.62	−2.05	0.049				
	Parietal (left)	0.00	−0.01	−0.01	0.994				
	Parietal (right)	0.04	0.41	0.59	0.563				
Post 30 min	Group: EG	0.06	0.10	0.62	0.540	1.27	6,32	0.299	0.04
	MMSE	0.08	0.30	1.70	0.098				
	Frontal (left)	0.01	0.09	0.25	0.803				
	Frontal (right)	−0.05	−0.67	−1.72	0.095				
	Parietal (left)	0.03	0.35	0.76	0.453				
	Parietal (right)	0.02	0.28	0.58	0.565				
Post 45 min	Group: EG	0.04	0.07	0.42	0.679	1.10	6,31	0.383	0.02
	MMSE	0.06	0.20	1.18	0.246				
	Frontal (left)	0.03	0.47	1.38	0.176				
	Frontal (right)	−0.04	−0.47	−1.42	0.164				
	Parietal (left)	0.01	0.12	0.28	0.783				
	Parietal (right)	0.01	0.11	0.24	0.811				

Note. *B* = non-standardized coefficients; *T* = *t*-test value; EG = experimental group; MMSE = Mini Mental State Examination. For the categorical variable group, the control group (CG) serves as reference category.

## Supplementary Materials

The following are available online at https://www.mdpi.com/2076-3425/10/11/813/s1, Table S1: Participant characteristics of the Experimental (EG) and control group (CG) for the fNIRS ANOVA, Table S2: Overview of all channels (i.e., source-detector combination) and their respective international 10-20 system position and corresponding ROI, Table S3: Average heart rate values for the experimental (EG) and control group (CG) at the start and end of each n-back task at time point pre (baseline) and follow-up measurements (post 15 min to post 45 min), Table S4: Time course of 0-back task performance for the experimental (EG) and control group (CG), Table S5: Time course of 1-back task performance for the experimental (EG) and control group (CG), Table S6: Repeated-measures ANOVA statistics for perceived difficulty, Table S7: Mean values and standard errors of cortical hemodynamic activity as measured by HBdiff in region (frontal, parietal) and hemisphere (left, right) during the 2-back task for the experimental (EG) and control group (CG), Table S8: Repeated-measures ANOVA statistics for fNIRS data.
