# Supplementary material for: Shedding Light on the Effects of Moderate Acute Exercise on Working Memory Performance in Healthy Older Adults: An fNIRS Study
Source: Brain Sci. 2020 Nov 3;10(11):813. doi: 10.3390/brainsci10110813 (PMC7693615; doi:10.3390/brainsci10110813)
Supplement: Supplementary file 1 [file brainsci-10-00813-s001.pdf]

brain sciences

Table S1. Participant characteristics of the experimental (EG) and control group (CG) for the fNIRS ANOVA.

	**EG** **(*n* = 16)**	**CG** **(*n* = 19)**	***p***
Age (range)	68.50 ± 3.44 (65–79)	69.42 ± 4.27 (64–79)	0.485
Gender (f ǀ m)	9 ǀ 7	8 ǀ 11	0.621
BMI (kg/m^2)	24.39 ± 2.36	24.92 ± 2.60	0.525
Education (years)	15.66 ± 2.44	15.74 ± 2.50	0.924
MMSE (sum score)	29.13 ± 0.99	28.89 ± 1.20	0.529
VO_2-peak (ml/kg/min)	25.91 ± 6.44	24.88 ± 6.84	0.652
Heat rate (bpm) at VO_2-peak	139.26 ± 19.45	138.42 ± 13.83	0.887
Watt (W) at VO_2-peak	135.25 ± 33.77	141.05 ± 42.90	0.657
Borg score (RPE) at VO_2-peak	12.13 ± 1.27	12.55 ± 1.70	0.413

Note. All values are given as mean (*M*) ± standard deviation (*SD*). Age = age in years; Gender = female ǀ male, BMI = Body Mass Index expressed in kg/m^2; Education = years of formal education; MMSE = sum score of the Mini Mental State Examination; VO_2-peak = peak oxygen uptake during the cardiovascular fitness test expressed in ml/kg/min; bpm = beats per minute; RPE = rate of perceived exertion according to Borg's 6-20 RPE scale.

Table S2. Overview of all channels (i.e., source-detector combinations) and their respective international 10-20 system position and corresponding ROI.

ROI	Channel	Source Number	Corresponding to 10–20 System	Detector Number	Corresponding to 10–20 System
Right DLPFC	1	S1	F1	D1	F3
	2	S2	AF3	D1	F3
	3	S2	AF3	D2	AF7
	4	S3	FC3	D1	F3
Left VLPFC	5	S3	FC3	D3	FC5
	6	S4	F5	D1	F3
	7	S4	F5	D2	AF7
	8	S4	F5	D3	FC5
	9	S4	F5	D4	F7
Right VLPFC	10	S5	F6	D5	F8
	11	S5	F6	D6	AF8
	12	S5	F6	D7	FC6
	13	S5	F6	D8	F4
	16	S7	FC4	D7	FC6
Right DLPFC	14	S6	AF7	D6	AF8
	15	S6	AF7	D8	F4
	17	S7	FC4	D8	F4
	18	S8	F2	D8	F4
Left IPL	19	S9	CP1	D9	CP3
	21	S10	CP5	D9	CP3
	22	S10	CP5	D11	P5
	23	S11	P3	D9	CP3
	25	S11	P3	D11	P5
Left SPL	20	S9	CP1	D10	P1
	24	S11	P3	D10	P1
Right IPL	29	S13	CP2	D13	CP4
	31	S14	CP6	D13	CP4
	32	S14	CP6	D15	P6
	33	S15	P4	D13	CP4
	35	S15	P4	D15	P6
Right SPL	34	S15	P4	D14	P2
	30	S13	CP2	D14	P2
none/excluded	26	S11	P3	D12	PO3
	27	S12	PO7	D11	P5
	28	S12	PO7	D12	PO3
	36	S15	P4	D16	PO4
	37	S16	PO8	D15	P6
	38	S16	PO8	D16	PO4

Note. ROI = region of interest. S = source; D = detector; DLPFC = dorsolateral prefrontal cortex; VLPFC = ventrolateral prefrontal cortex; IPL = inferior parietal lobe; SPL = superior parietal lobe.

Table S3. Average heart rate values for the experimental (EG) and control group (CG) at the start and end of each n-back task at time point pre (baseline) and follow-up measurements (post 15 min to post 45 min).

Time Point	Group	Time Point	*M* (bpm)	*SD* (bpm)	*Min* (bpm)	*Max* (bpm)
Pre	EG	start	69.37	10.76	48	93
	CG	start	72.43	9.21	54	96
	EG	end	67.84	9.46	47	82
	CG	end	70.43	10.10	49	94
Post 15 min	EG	start	77.42	13.26	49	99
	CG	start	68.30	8.65	48	85
	EG	end	74.47	13.24	48	97
	CG	end	67.87	9.06	49	86
Post 30 min	EG	start	74.00	12.09	50	96
	CG	start	67.22	7.83	49	79
	EG	end	71.42	11.04	47	89
	CG	end	67.13	8.56	48	81
Post 45 min	EG	start	70.11	11.43	47	92
	CG	start	65.70	7.53	49	82
	EG	end	71.21	11.27	46	89
	CG	end	66.04	7.76	50	81

Note. *M* = mean; *SD* = standard deviation; *Min* = minimum; *Max* = maximum; bpm = beats per minute.

Table S4. Time course of 0-back task performance for the experimental (EG) and control group (CG).

	Time Point	EG (n = 19)	CG (n = 23)
		**0-back**	**0-back**
RT per trial (ms)	Pre	646.31 ± 42.63	614.75 ± 45.09
	Post 15 min	547.90 ± 21.96	559.08 ± 20.64
	Post 30 min	550.42 ± 21.27	557.51 ± 21.65
	Post 45 min	559.43 ± 23.25	556.26 ± 19.13
ACC (%)	Pre	93.27 ± 2.32	96.15 ± 1.89
	Post 15 min	98.79 ± 0.42	98.16 ± 0.59
	Post 30 min	98.58 ± 0.73	99.33 ± 0.31
	Post 45 min	98.58 ± 0.67	98.83 ± 0.70
RCS	Pre	1.56 ± 0.10	1.69 ± 0.08
	Post 15 min	1.85 ± 0.07	1.81 ± 0.07
	Post 30 min	1.83 ± 0.06	1.84 ± 0.07
	Post 45 min	1.82 ± 0.07	1.83 ± 0.07
Perceived difficulty	Pre	9.21 ± 0.35	9.13 ± 0.45
	Post 15 min	8.42 ± 0.34	8.35 ± 0.38
	Post 30 min	8.58 ± 0.35	8.04 ± 0.37
	Post 45 min	8.79 ± 0.42	8.39 ± 0.49

Note. *N* = 42. All values are given as mean (*M*) ± one standard error of the mean (*SE*). RT = reaction time. ACC = accuracy. RCS = rate-correct score.

Table S5. Time course of 1-back task performance for the experimental (EG) and control group (CG).

	Time Point	EG (n = 19)	CG (n = 23)
		1-back	1-back
RT per trial (ms)	Pre	736.16 ± 43.77	765.83 ± 48.04
	Post 15 min	658.87 ± 33.36	704.49 ± 40.56
	Post 30 min	653.41 ± 31.31	640.41 ± 36.16
	Post 45 min	635.75 ± 29.64	654.10 ± 36.26
ACC (%)	Pre	92.31 ± 1.38	92.98 ± 1.62
	Post 15 min	96.96 ± 0.69	96.32 ± 0.89
	Post 30 min	97.98 ± 0.54	98.33 ± 0.47
	Post 45 min	98.18 ± 0.74	97.49 ± 0.79
RCS	Pre	1.33 ± 0.08	1.31 ± 0.08
	Post 15 min	1.54 ± 0.08	1.48 ± 0.07
	Post 30 min	1.56 ± 0.07	1.62 ± 0.07
	Post 45 min	1.60 ± 0.07	1.57 ± 0.07
Perceived difficulty	Pre	11.00 ± 0.42	10.35 ± 0.38
	Post 15 min	10.53 ± 0.39	9.70 ± 0.39
	Post 30 min	10.32 ± 0.51	9.65 ± 0.44
	Post 45 min	10.32 ± 0.49	9.87 ± 0.49

Note. N = 42. All values are given as mean (M) ± one standard error of the mean (SE). RT = reaction time. ACC = accuracy. RCS = rate-correct score.

Table S6. Repeated-measures ANOVA statistics for perceived difficulty.

ANOVA				
**Effect**	***F* ratio**	**df**	p	η_{ges}^2
G	0.94	1,40	0.338	0.01
T	6.64	2.17,86.81	0.002 **	0.02
C	187.45	1.54,61.48	<0.001 ***	0.53
G × T	0.09	2.17,86.81	0.925	<0.01
G × C	0.33	1.54,61.48	0.660	<0.01
T × C	0.62	4.61,184.37	0.673	<0.01
G × T × C	0.78	4.61,184.37	0.556	<0.01

Note. N = 42. ANOVA = analysis of variance; C = Condition; G = Group; T = Time. ** $p < .01$ ***$p < .001$.

Table S7. Mean values and standard errors of cortical hemodynamic activity as measured by HBdiff in region (frontal, parietal) and hemisphere (left, right) during the 2-back task for the experimental (EG) and control group (CG).

			**EG (*n* = 16)**		**CG (*n* = 19)**	
**Time Point**	**Hemisphere**	**Region**	***M***	***SE***	***M***	***SE***
Pre	Left	Frontal	0.18	1.05	1.94	0.62
	Left	Parietal	1.17	1.36	2.24	0.87
	Right	Frontal	0.88	0.91	1.81	0.82
	Right	Parietal	1.20	1.25	2.12	1.14
Post 15 min	Left	Frontal	0.60	0.67	1.88	0.73
	Left	Parietal	1.03	1.36	2.74	0.83
	Right	Frontal	0.63	0.77	1.56	0.62
	Right	Parietal	1.11	1.18	2.51	0.82
Post 30 min	Left	Frontal	0.72	0.76	1.42	0.77
	Left	Parietal	1.85	1.08	2.40	0.60
	Right	Frontal	1.12	0.83	1.58	0.79
	Right	Parietal	1.54	1.01	2.06	0.68
Post 45 min	Left	Frontal	1.24	1.48	0.76	0.88
	Left	Parietal	3.29	1.10	1.87	0.80
	Right	Frontal	0.56	1.33	0.92	0.77
	Right	Parietal	2.65	1.04	1.72	0.72

Note. *M* = mean; *SE* = standard error.

Table S8. Repeated-measures ANOVA statistics for fNIRS data.

	**ANOVA**			
**Effect**	***F* ratio**	**df**	***p***	η_{ges}^2
G	0.50	1,33	0.485	0.01
T	0.03	2.69,88.80	0.989	<0.01
H	0.20	1,33	0.659	<0.01
R	4.59	1,33	0.040*	0.01
G × T	0.88	2.69,88.80	0.444	0.01
G × H	0.03	1,33	0.854	<0.01
G × R	0.11	1,33	0.743	<0.01
T × H	1.08	2.73,90.24	0.358	0.36
T × R	1.66	2.03,66.70	0.197	<0.01
H × R	1.04	1,33	0.315	<0.01
G × T × H	1.97	2.73,90.24	0.129	<0.01
G × T × R	0.88	2.03,66.70	0.420	<0.01
G × H × R	0.08	1,33	0.778	<0.01
T × H × R	0.92	2.66,87.82	0.426	<0.01
G × T × H × R	0.51	2.66,87.82	0.653	<0.01

Note. $n = 35$. ANOVA = analysis of variance; G = Group; H = Hemisphere; R = Region; T = Time. *$p < 0.05$.
